# Immunogenic cell death-related classifications guide prognosis and immunotherapy in osteosarcoma

**DOI:** 10.1038/s41598-023-35745-w

**Published:** 2023-06-05

**Authors:** Yuan Zong, Yu Cao, Ding Zhang, Xiaoqing Guan, Fengyi Zhang, Zhubin Shen, Fei Yin

**Affiliations:** 1 Department of Orthopedic Surgery, China-Japan Union Hospital, Jilin University, Changchun, Jilin People’s Republic of China; 2 Department of Orthodontics, Hospital of Stomatology, Jilin University, No. 1500 Qinghua Street, Changchun, 130021 Jilin People’s Republic of China

**Keywords:** Bone cancer, Cancer microenvironment, Tumour immunology, Molecular biology, Cell death and immune response, Tumour immunology

## Abstract

Immunogenic cell death (ICD) is a form of cell death that stimulates the immune system to produce an immune response by releasing tumour-associated antigens and tumour-specific antigens and is considered to play an important role in tumour immunotherapy. In the present study, we identified two ICD-related subtypes in osteosarcoma (OS) by consensus clustering. The ICD-low subtype was associated with favourable clinical outcomes, abundant immune cell infiltration, and high activity of immune response signalling. We also established and validated an ICD-related prognostic model, which could not only be used to predict the overall survival of OS patients but was also found to be closely related to the tumour immune microenvironment of OS patients. Overall, we established a new classification system for OS based on ICD-related genes, which can be used to predict the prognosis of OS patients and to select appropriate immunotherapy drugs.

## Introduction

During chemotherapy for tumours, drugs can inhibit tumour growth by inducing apoptosis or another programmed death mode of tumour cells^1^. Apoptotic tumour cells have long been believed to be nonimmunogenic and immune tolerant^2,3^, but a large number of recent studies have shown that some apoptotic tumour cells are also immunogenic, that is, immunogenic cell death (ICD)^4,5^. ICD is a specific variant of regulated cell death (RCD) driven by stress^6^. ICD stimulates the immune system to produce an immune response by releasing tumour-associated antigens (TAAs) and tumour-specific antigens (TSAs) and is characterized by the release and/or increased expression of damage-associated molecular patterns (DAMPs), precursor antigens, inflammatory cytokines, and inflammatory mediators^7,8^. Therefore, whether it can induce the immunogenic death of tumour cells is one of the important factors affecting the therapeutic effect.

Osteosarcoma (OS) is the most common malignant bone tumour, and its high-incidence population is mainly teenagers, accounting for approximately 5% of the total number of paediatric tumours^9,10^. Although great advances have been made in the 5-year survival rate of OS patients, the outcomes of advanced OS patients remain unsatisfactory, which results from chemotherapy resistance and cancer cell metastasis^11,12^. Chemotherapy is a conventional treatment for OS, and it plays an important role in the treatment of OS patients with local bone pain and recurrence as well as those who cannot be operated on or refuse surgery^13,14^. However, the sensitivity of OS cells to chemotherapy drugs is poor, so some patients with OS develop chemotherapy resistance, which greatly affects the effect of chemotherapy and has become a key factor affecting the treatment efficacy and prognosis of OS patients^13,14^. Therefore, further study on the prediction of the sensitivity to chemotherapy drugs in OS cells is urgently needed, which is significant for improving the overall survival of patients with OS.

Recently, with the rapid development of bioinformatics technology, bioinformatics has become increasingly popular in studying the molecular mechanism of diseases and discovering disease-specific biomarkers, which are increasingly used for the accurate diagnosis and treatment of diseases. Some recently published articles describe the establishment of new cancer classification systems based on ICD-related classification by analysing the transcriptome expression profile dataset of cancer tissues in public databases, including head and neck squamous cell carcinoma^15^ and intracranial aneurysms^16^. This ICD-related classification can be used to predict the prognosis and drug sensitivity of cancer patients.

In this study, we aimed to identify an ICD-related gene-based risk assessment model that is beneficial for assessing the immune microenvironment, overall survival, and response to treatment in patients with OS, which can help physicians make important decisions about the treatment of OS patients.

## Results

### Consensus clustering identifies two subtypes associated with ICD

**Figure 1:**
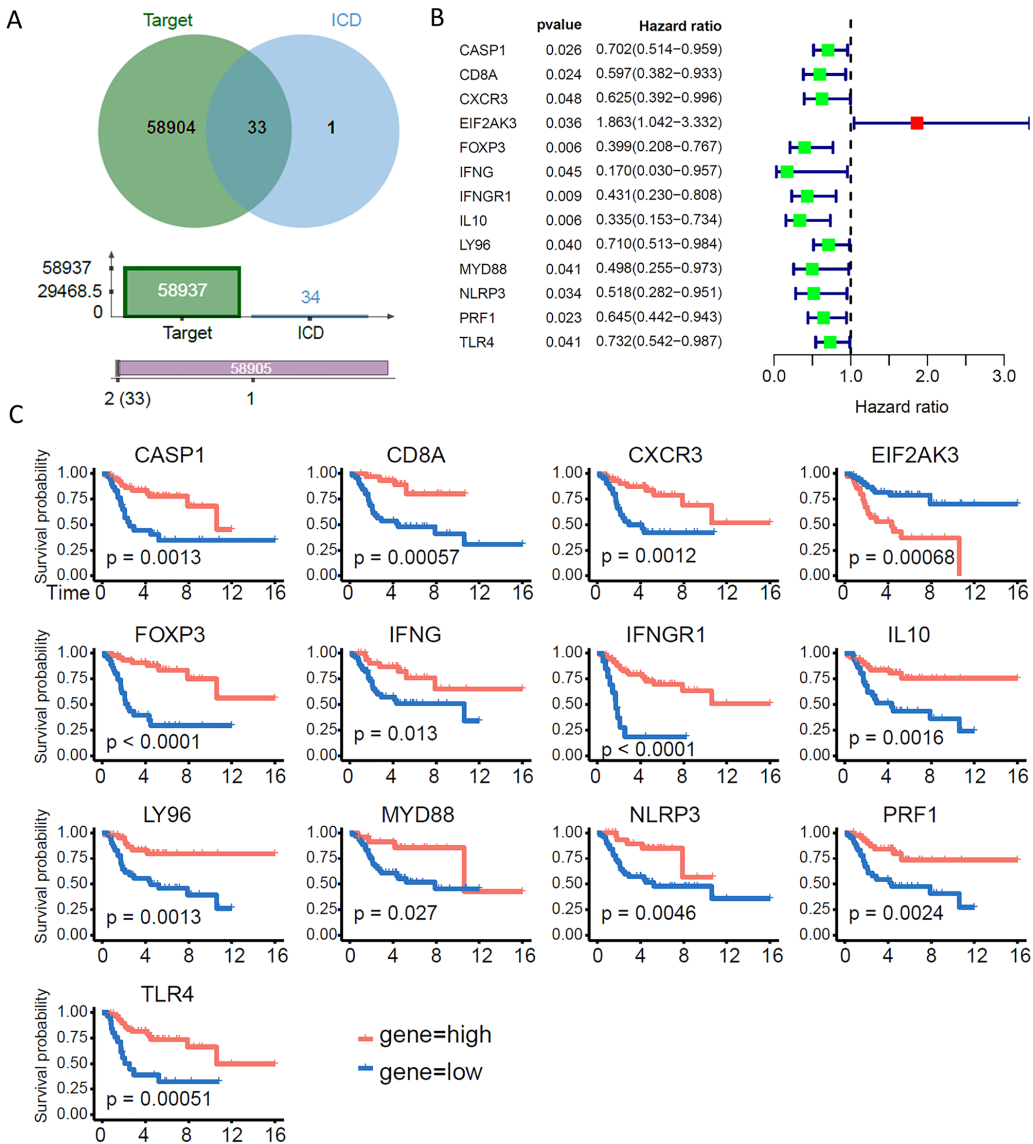
Identification of 13 ICD-related genes associated with OS patient prognosis. (**A**) Venn diagram showing that there were 33 intersecting genes between the OS-expressed genes in the Target database and the 34 known ICD-related genes. (**B**) Of the 33 intersecting genes, 13 prognosis-related genes were screened out by univariate Cox analysis. (**C**) Kaplan‒Meier survival analysis curves of 13 genes in patients with OS.

**Figure 2:**
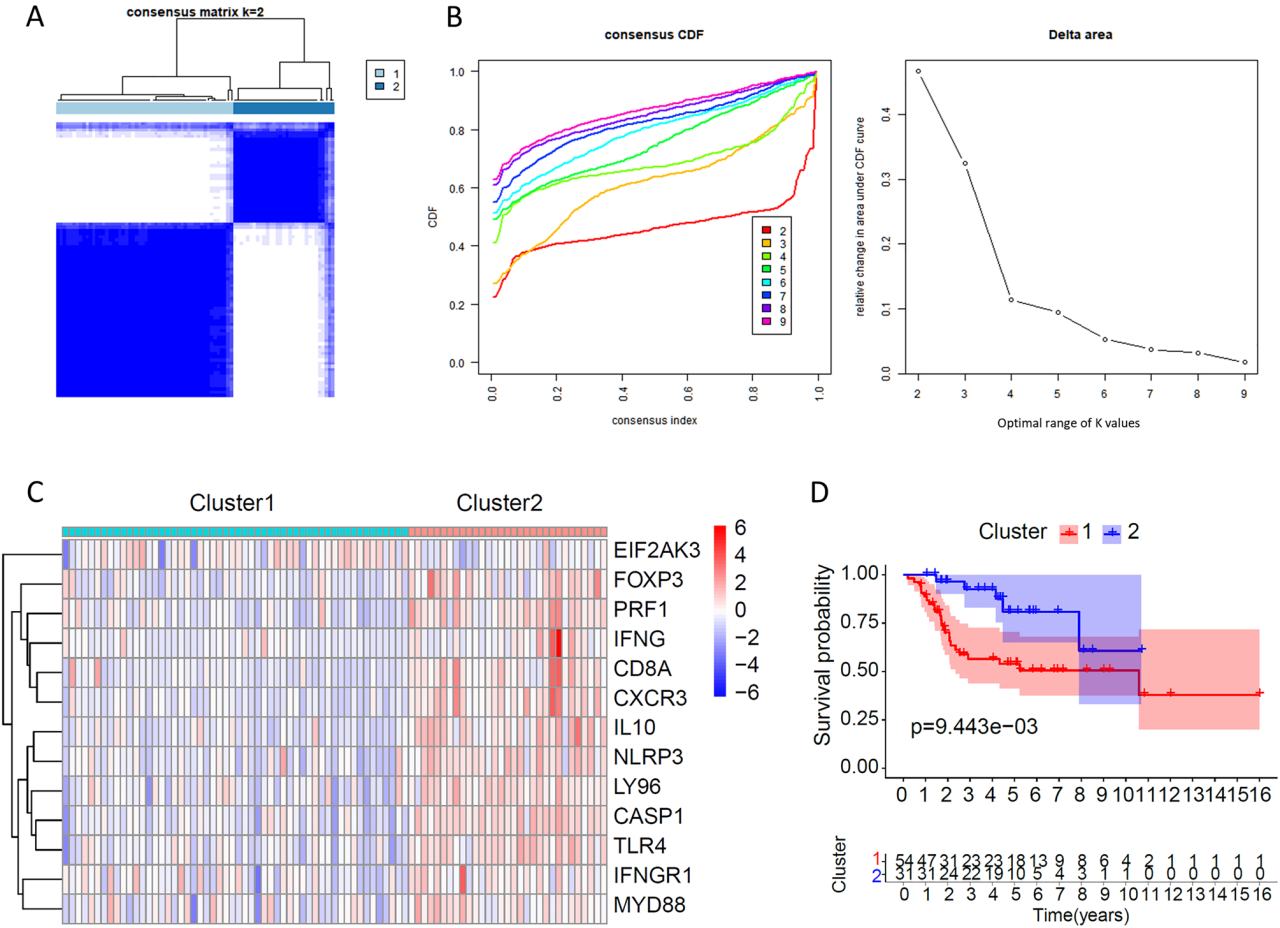
Consensus clustering was used to identify ICD-related subtypes. We plotted the results with the R software (version 4.2.2 https://cran.r-project.org/src/base/R-4/). (**A**) Heatmap depicts consensus clustering solution (k = 2) for 13 genes in 85 OS samples. (**B**) Delta area curve of consensus clustering indicates the relative change in the area under the cumulative distribution function (CDF) curve for k = 2 to 10. (**C**) Heatmap of the expression of 13 ICD-related genes in different subtypes. Red and blue represent high and low expression, respectively. (**D**) Kaplan–Meier curves of OS in the ICD-high and ICD-low subtypes.

Thirty-four ICD-related genes have been reported by many studies, and we cross-checked the abnormally expressed genes of OS samples in the Target database with these 34 ICD-related genes. As shown in Fig. 1A, the Venn diagram showed that there were 33 intersecting genes between the OS-expressed genes. Next, prognostic evaluation of the 33 identified ICD-related genes by univariate Cox analysis showed that 13 genes were associated with the prognosis of OS patients (Fig. 1B), including CASP1, CD8A, CXCR3, EIF2AK3, FOXP3, IFNG, IFNGR1, IL-10, LY96, MYD88, NLRP3, PRF1 and TLR4. In addition, we performed Kaplan‒Meier survival analysis of the 13 ICD-related genes identified in OS patients and found that they were all significantly related to the survival of OS patients (Fig. 1C). We next identified OS clusters associated with ICD using consensus clustering. Two clusters in the target cohort were identified as having distinct ICD gene expression patterns after k-means clustering (Fig. 2A,B). In conclusion, clusters 1 and 2 showed low (ICD-low subtype) and high (ICD-high subtype) ICD-related gene expression levels, respectively (Fig. 2C). Moreover, the survival analysis results showed that the ICD-high subtype had a better prognosis (Fig. 2D).

### Identification of differentially expressed genes and signalling pathways

**Figure 3:**
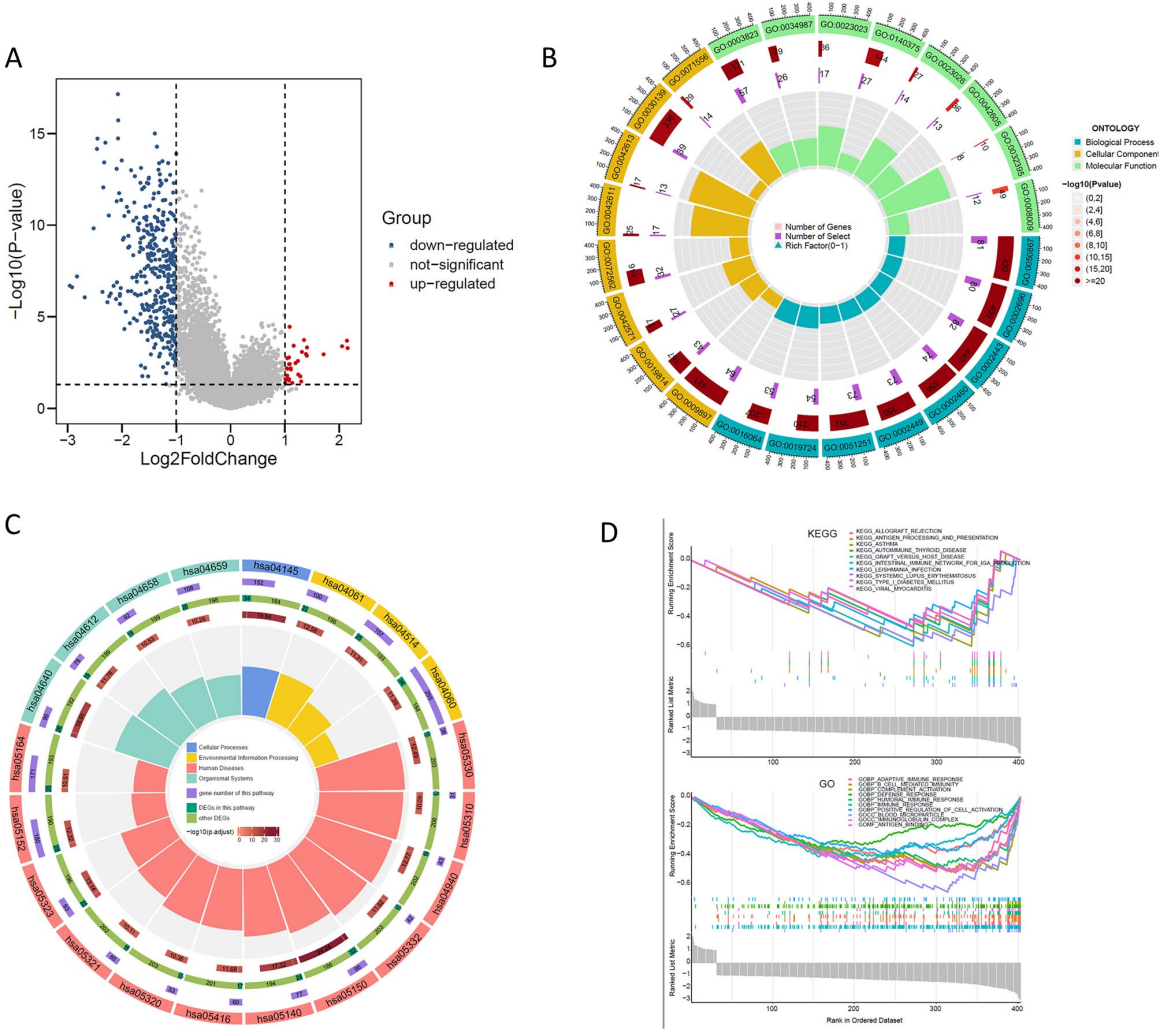
Identification of DEGs and signalling pathways in different ICD subtypes. We plotted the result with the R software (version 4.2.2 https://cran.r-project.org/src/base/R-4/). (**A**) Volcano plot shows the quantified DEG distribution between the ICD-high and ICD-low subtypes (log2-fold change > 1 or log2-fold change < − 1, P < 0.05). (**B**) Circos plot shows the GO signalling pathway enrichment analysis results. (**C**) Circos plot presents the KEGG pathway enrichment analysis results. The size of the dot represents the gene count, and the colour of the dot represents the − log10 (p. adjust value) value. (**D**) GSEA identified potential signalling pathways between the ICD-high and ICD-low subtypes.

We identified differentially expressed genes (DEGs) and critical pathways for the ICD-low and ICD-high subtypes. Figure 3A shows the 414 DEGs that were identified between the two subtypes. Functional enrichment analysis indicated that the DEGs were enriched in immune-related activity, including cytokine‒cytokine receptor interaction, Th17 cell differentiation, Th1 and Th2 cell differentiation, adaptive immune response, B-cell-mediated immunity, and immune receptor activity (Fig. 3B,C). Furthermore, we performed gene set enrichment analysis (GSEA) to identify relevant signalling pathways for the two subtypes. GSEA revealed that the DEGs were related to immune pathways, such as B-cell-mediated immunity, defence response, human immune response, lymphocyte-mediated immunity, and antigen processing and presentation (Fig. 3D).

### Tumour microenvironment landscapes of the ICD-high and ICD-low subtypes

**Figure 4:**
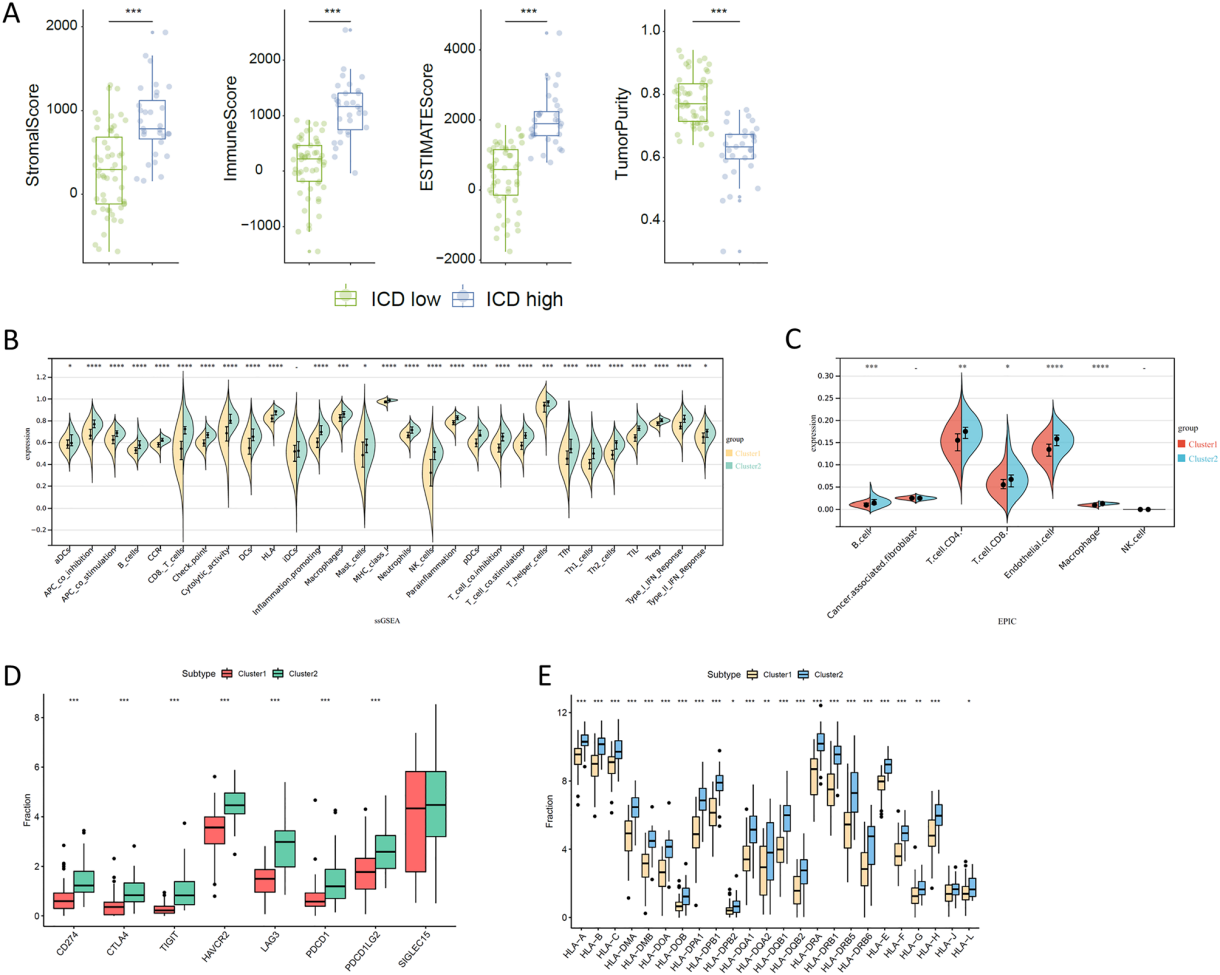
Immune microenvironment of different ICD subtypes. (**A**) Box plot showing the stromal score, immune score and ESTIMATE score of different ICD subtypes analysed by ESTIMATE. *** indicates P < 0.001. The stromal score captures the presence of stroma in tumour tissue, the immune score represents the infiltration of immune cells in tumour tissue, the ESTIMATE score indicates tumour purity, and the Tumor purity was calculated from the three scores mentioned above. (**B**,**C**) ssGSEA (**B**) and EPIC (**C**) analyses showed significantly different immune cells between different ICD subtypes. ^–^P > 0.05, *P < 0.05, **P < 0.01 and ***P < 0.001. (**D**) Box plots present the differential expression of multiple immune checkpoints (**D**) and HLA genes (**E**) between different ICD subtypes. *P < 0.05, **P < 0.01 and ***P < 0.001.

ICD affects the activation of certain antitumour immune responses. We analysed the composition of the tumour microenvironment in the ICD-low and ICD-high subtypes, and the results showed that compared with the ICD-low subtype, the ICD-high subtype had a higher stromal score, immune score, and ESTIMATE score but a lower tumour purity (Fig. 4A). Then, we assessed differences in the immune infiltration of multiple immune cells between the two subtypes using single-sample GSEA (ssGSEA) and EPIC methods and found that patients with the ICD-high subtype had higher proportions of B cells, CD4 T cells, CD8 T cells, endothelial cells and macrophages (Fig. 4B,C). In addition, many immune checkpoints (Fig. 4D) and human leukocyte antigen (HLA) genes (Fig. 4E) were upregulated in the ICD-high subtype, suggesting that the ICD-high subtype is associated with the immunothermal phenotype.

### Construction and validation of the ICD risk signature

**Figure 5:**
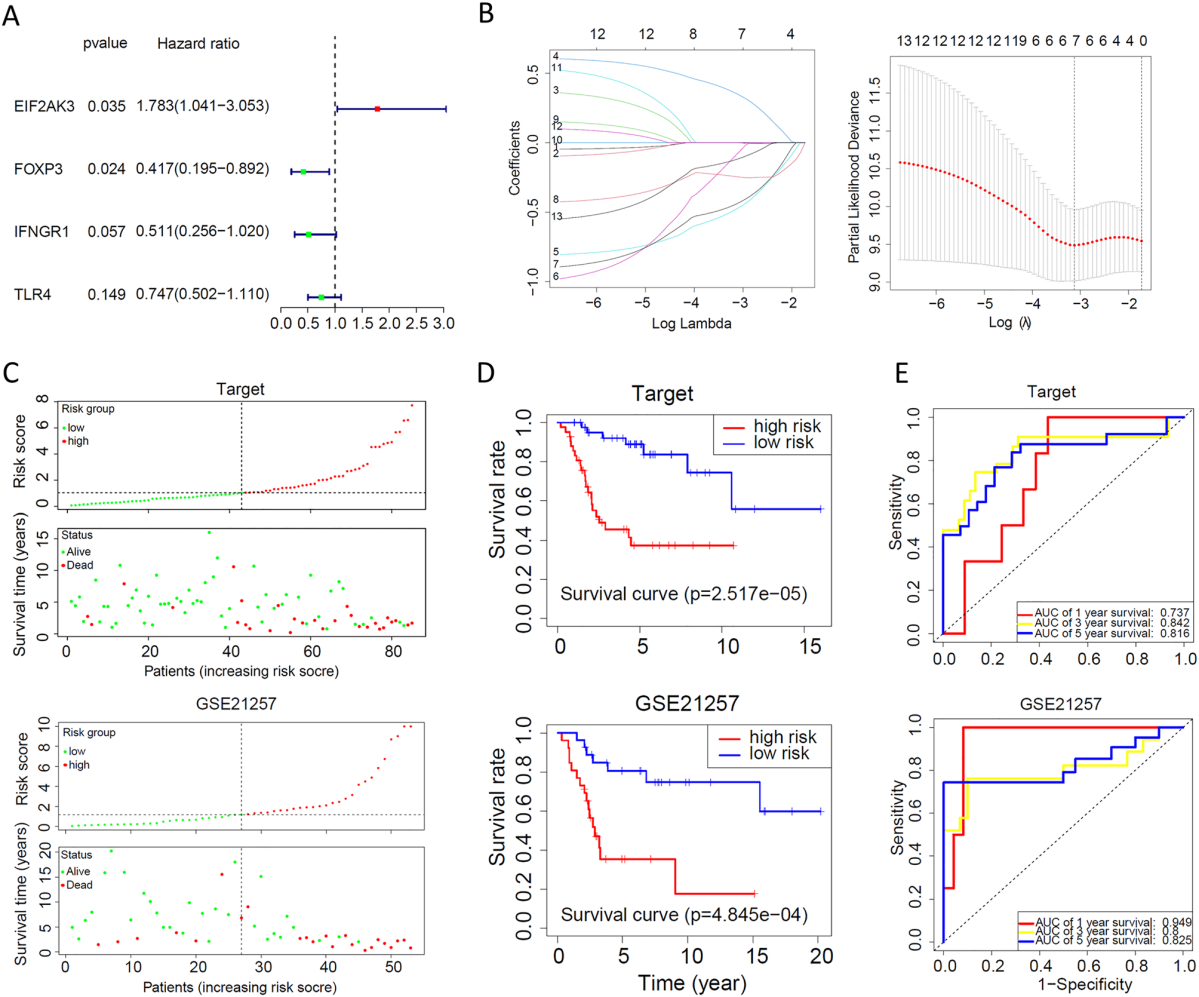
Construction and verification of the ICD risk signature. (**A**) Four ICD-related genes were found to be significantly associated with OS in univariate Cox analysis. (**B**) LASSO Cox analysis identified 12 genes most associated with OS in the Target and GEO datasets. (**C**) Risk score distribution and survival status for each patient in the Target and GEO databases. (**D**) Kaplan‒Meier analysis demonstrates the prognostic significance of the risk model in the Target and GSE21257 cohorts. (**E**) ROC demonstrates that the diagnostic performance of the risk model for prognosis is good in the Target and GSE21257 cohorts.

The results of univariate Cox analysis showed that a total of 4 ICD-related genes were significantly associated with OS (Fig. 5A). Twelve ICD-related genes were tested and selected for the prediction model in the least absolute shrinkage and selection operator (LASSO) regression analysis (Fig. 5B). In addition, we investigated the relationship between the risk score and the survival status of OS patients and found that low-risk patients had a better survival status (Fig. 5C). Both the target cohort and the Gene Expression Omnibus (GEO) cohort showed that the survival rate of the low-risk group was significantly higher than that of the high-risk group (Fig. 5D). In addition, receiver operating characteristic (ROC) curve analysis showed that the diagnostic performance of the risk model for prognosis was good in the target cohort and the GEO cohort (Fig. 5E).

### Correlation of the ICD risk signature with the OS tumour microenvironment

**Figure 6:**
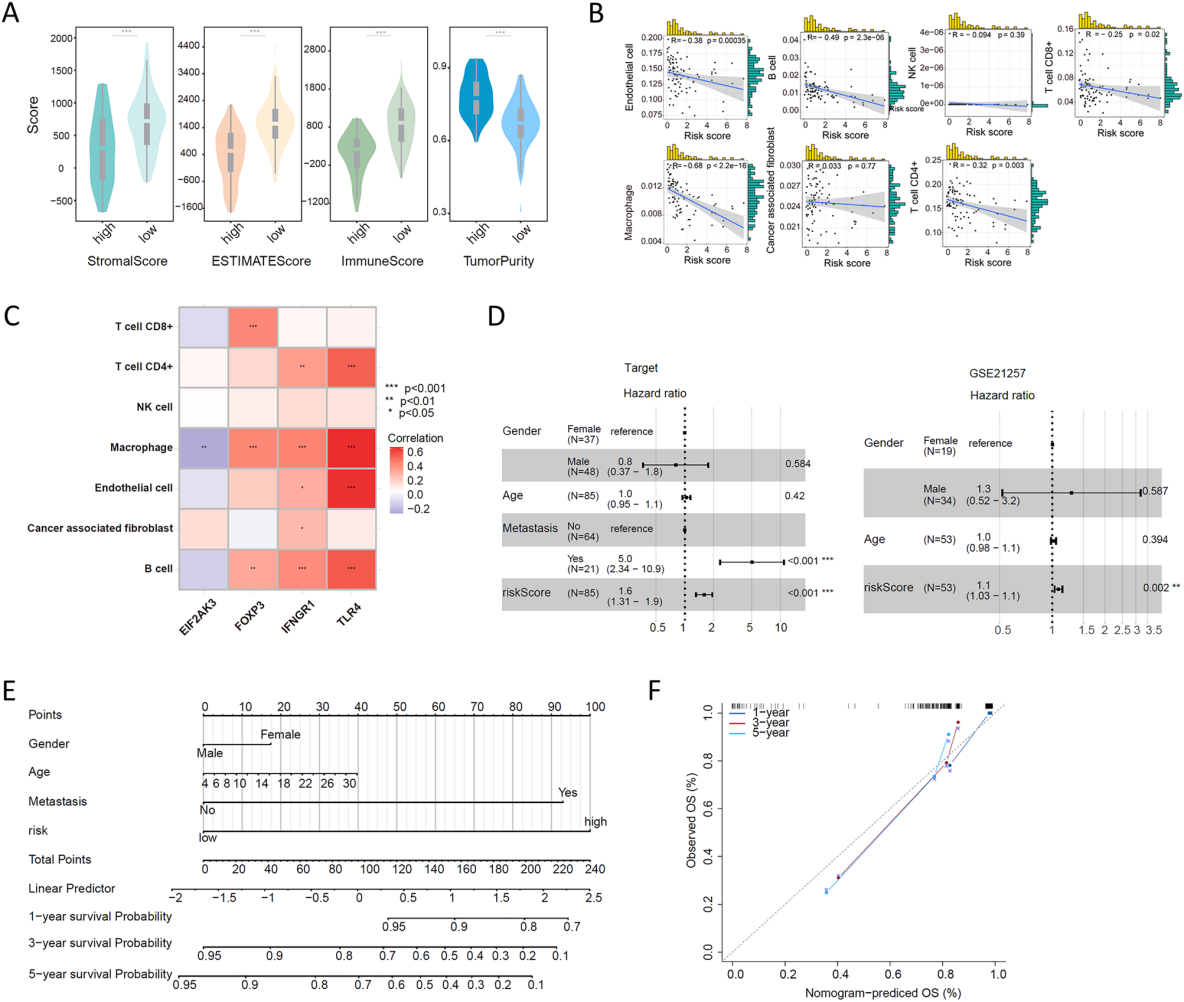
Correlation of the ICD risk signature with the OS tumour microenvironment. (**A**) Violin plots showing the stromal score, immune score, ESTMATE score and tumour purity in the high-risk and low-risk groups. ***P < 0.001. (**B**,**C**) Scatter plot (**B**) and heatmap (**C**) showing the correlation of the risk score with immune cell infiltration in the OS tumour tissue microenvironment. (**D**,**E**) Results of univariate and multivariate Cox analyses (**D**) and nomogram (**E**) assessing the independent prognostic value of the ICD risk signature in OS patients. (**F**) The calibration curve shows that the performance of the nomogram is good.

**Figure 7:**
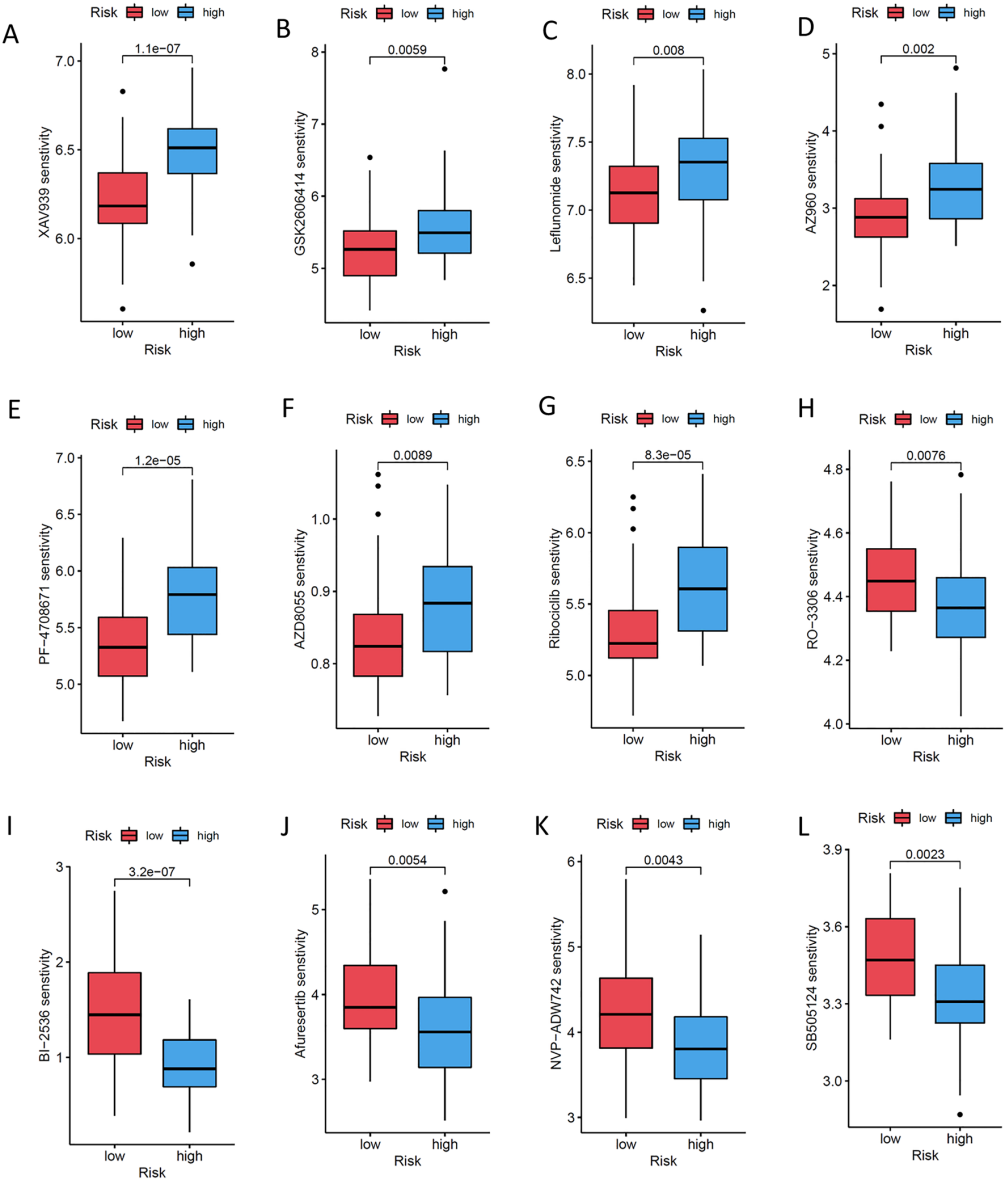
Boxplots showing the association of the ICD risk score with the response to different drug treatments. (**A**) XAV939; (**B**) GSK2606414; (**C**) leflunomide; (**D**) AZ960; (**E**) PF-4708671; (**F**) AZD8055; (**G**) ribociclib; (**H**) RO-3306; (**I**) BI-2536; (**J**) afuresertib; (**K**) NVP-ADW742; and (**L**) SB505124.

We analysed the composition of the tumour microenvironment in the high-risk and low-risk groups, and the results showed that compared with the high-risk group, the low-risk group had higher stromal scores, immune scores and ESTIMATE scores but lower tumour purity (Fig. 6A). We analysed the correlation of the ICD risk signature with the OS tumour microenvironment and found that the risk score was negatively correlated with endothelial cells, B cells, CD4 T cells, CD8 T cells, and macrophages (Fig. 6B). We also analysed the correlation between the expression of ICD-related genes and these immune cells in the risk model, and the results showed that the expression of the high-risk gene EIF2AK3 was negatively correlated with the degree of macrophage infiltration, while the expression of the low-risk genes FOXP3, IFNGR1 and TLR4 was positively correlated with these immune cells (Fig. 6C). The results of univariate and multivariate Cox analyses showed that the risk score could be used as an independent predictor of prognosis in OS patients (Fig. 6D–F). Finally, we compared the sensitivity of OS patients in the high-risk and low-risk groups to immunotherapy drugs. The results showed that the low-risk group patients had better sensitivity and lower half-maximal inhibitory concentration (IC50) values for XAV939 (Fig. 7A), GSK2606414 (Fig. 7B), leflunomide (Fig. 7C), AZ960 (Fig. 7D), PF-4708671 (Fig. 7E), AZD8055 (Fig. 7F) and ribociclib (Fig. 7G), while the high-risk group patients had better sensitivity and lower IC50 values for RO-3306 (Figure 7H), BI-2536 (Fig. 7I), afuresertib (Fig. 7J), NVP-ADW742 (Fig. 7K) and SB505124 (Fig. 7L).

## Discussion

OS has the highest incidence among primary malignant bone tumours, but its overall incidence is low (4–5/million)^17,18^. Although OS has lower morbidity and mortality rates, it is worth noting that more than half of OS patients die from tumour cell metastasis and chemotherapy resistance^19,20^. Chemotherapy is the main treatment for OS and can kill metastatic cancer cells, but OS cells are less sensitive to chemotherapy drugs, and many patients develop resistance to chemotherapy^19,20^. Therefore, new treatment protocols are important for OS patients. Recently, an increasing number of studies have confirmed that ICD is expected to provide new ideas and strategies for antitumour immunotherapy due to its characteristics of immunogenicity, immune activation in tumours, and release of multiple tumour antigens.

In 2020, a new drug, belantamab mafodotin, developed based on the definition of ICD was approved by the FDA for the treatment of adult patients with relapsed or refractory multiple myeloma, indicating that ICD research is of great significance for the development of new drugs for the treatment of OS^21,22^. In this study, we identified two ICD-related subtypes in OS by consensus clustering and found that the ICD-low subtype is associated with favourable clinical outcomes, abundant immune cell infiltration, and high activity of immune response signalling. In addition, we established and validated an ICD-related prognostic model, which could not only be used to predict the overall survival of OS patients but was also found to be closely related to the tumour immune microenvironment of OS patients.

When tumour cells die due to external stimuli, the process of transforming from nonimmunogenic to immunogenic to mediate the body's antitumour immune response is called ICD^23,24^. When tumour cells develop ICD, they produce a series of signalling molecules called DAMPs, mainly involving calreticulin exposed on the cell surface, high mobility group box 1 (HMGB1) secreted by tumour cells to the outside world, ATP molecules released by cells, and heat shock proteins (HSP70 and HSP90)^6^. DAMPs released during the ICD process can bind to pattern recognition receptors (PRRs) on the surface of DCs to initiate a series of cellular responses that ultimately activate innate and adaptive immune responses^7,8^. ICD can be caused by a variety of different stressors, including but not limited to (1) intracellular pathogens; (2) traditional chemotherapy drugs such as anthracyclines, DNA damaging agents and proteasome inhibitors; (3) targeted anticancer drugs; and (4) a variety of physical therapies^8,25^. Based on this evidence, our study identified two ICD subgroups by consensus clustering, and the ICD-low subgroup was associated with the immune-hot phenotype, while the ICD-high subgroup was associated with the immune-cold phenotype.

It has been found that chemotherapy drugs, radiotherapy and photodynamic therapy can induce the immunogenic death of tumour cells, and an increasing number of chemotherapeutic drugs will be found to induce the immunogenic death of tumour cells as research progresses^26,27^. Taking full advantage of these treatments will lead to more effective treatments for cancer. In the present study, we found that OS patients with different risk scores had different susceptibilities to various drugs, which indicated that our OS risk score model based on ICD could help clinicians select optimal therapeutic drugs.

Overall, our study highlights that OS classification based on ICD-related genes is closely related to changes in the immune tumour microenvironment, and these observations can be used not only to predict the clinical prognosis of OS patients but also to help clinicians choose appropriate treatment protocols.

## Methods

### Database

The RNA sequencing (RNA-seq) transcriptomic information and clinical information of 85 osteosarcoma (OS) patients from the Target database (https://ocg.cancer.gov/programs/target) were used as the training set. The RNA-seq and clinical information of 53 OS patients (GSE21257) from the Gene Expression Omnibus (GEO, https://www.ncbi.nlm.nih.gov/geo/query/acc.cgi?acc=GSE21257) database were used as the validation set.

### Statistical analysis

All data processing and analysis was done using R software (version 4.2.2). The *t* test was used to compare the two groups with continuous variables and to assess the statistical significance of normally distributed variables. The independent and the differences between non-normally distributed variables were analysed using the Mann–Whitney *U* test (i.e. the Wilcoxon rank sum test). For comparison and analysis of statistical significance between two groups of categorical variables, the chi-square test or Fisher's exact test was used. Correlation coefficients between different genes were calculated using Pearson correlation analysis. The *t* test was used to compare the values of the mean between two groups of samples. P < 0.05 was considered statistically significant.

### Consensus clustering

Immunogenic cell death (ICD)-related genes were coclustered using the ConsensusClusterPlus function in R to identify ICD molecular subtypes, followed by evaluation of the ideal number of clusters between K = 2–10 to ensure stability of the results after no less than 1000 replicates.

### Screening of differentially expressed genes

We used the R/Bioconductor package limma (https://bioconductor.org/packages/release/bioc/html/limma.html) to analyse the data and plotted the results (volcano maps) with the R package ggplot (https://cran.r-project.org/web/packages/ggplot2/index.html). The differentially expressed gene (DEG) screening criteria were as follows: adjusted P < 0.05 and |fold change| > 1.

### Functional enrichment analysis

The R/Bioconductor software package ClusterProfiler (https://bioconductor.org/packages/release/bioc/html/clusterProfiler.html) was used to perform Gene Ontology (GO) annotation and Kyoto Encyclopedia of Genes and Genomes (KEGG) pathway enrichment analysis of the DEGs obtained in the above steps. P < 0.05 indicated a significant difference.

### Construction of a prognosis-related gene model

Risk values were calculated for all patients based on a combination of the gene expression levels and their respective coefficients obtained from multivariate Cox regression analysis, namely, the risk score (RS). We calculated the RS of each patient according to the above formula and divided the patients into high-risk and low-risk groups using the median RS as the cut-off point.

### Independence of the prognostic model from other clinical features

To further determine whether the prognostic model was independent of other clinical characteristics, such as age, sex, and metastasis, we assessed the RS model for OS patients using univariate and multivariate Cox regression analyses. To further illustrate the relationship between the different variables, we also plotted a nomogram and its associated calibration curve. All tests were statistically analysed using R language software, and P < 0.05 indicated a significant difference.

### ESTIMATE algorithm to assess the tumour immune microenvironment

ESTIMATE (Estimation of STromal and Immune cells in MAlignant Tumor tissues using Expression data) (https://bioinformatics.mdanderson.org/estimate/index.html) is a tool for predicting tumour purity and the presence of infiltrating stromal/immune cells in tumour tissues using gene expression data^28^. We evaluated the tumour immune microenvironment in patients with OS from the Target dataset using the computational methods provided on this website. The stromal score captures the presence of stroma in tumour tissue, the immune score represents the infiltration of immune cells in tumour tissue, the ESTIMATE score indicates tumour purity, and the Tumor purity was calculated from the three scores mentioned above.

### EPIC

EPIC (https://epic.gfellerlab.org/) is a method that is used to calculate the ratio of immune cells to cancer cells from a large amount of tumour gene expression data^29^. This is done by fitting gene expression reference profiles from the main non-malignant cell types and simultaneously accounting for an uncharacterized cell type without prior knowledge about it (e.g. cancer cells in solid tumors samples). We used the EPIC algorithm to calculate the degree of immune cell infiltration in the osteosarcoma microenvironment and to evaluate the association of the constructed ICD gene with immune cells.

### Evaluation of drug sensitivity

The training and test expression data were quantile normalized separately using the R package pRRophetic (http://genemed.uchicago.edu/,pgeeleher/pRRophetic) and then combined by normalizing the mean and variance of each gene using an empirical Bayesian approach. Removal of genes with low variability between samples. A ridge regression model is fit to the training expression data using all remaining genes as predictors and the drug sensitivity (IC50) values (of the drug of interest) as the outcome variable. This model was applied to the processed, standardized, filtered clinical tumour expression data, yielding a drug sensitivity estimate for each patient. Through this algorithm, we screened out sensitive chemotherapy drugs that can be used to treat patients with osteosarcoma.

### Survival analysis

Kaplan‒Meier (KM) analysis was performed using the survminer and survival packages in R to compare overall survival in patients with OS in different groups, including the ICD-high and ICD-low groups and the high-risk score and low-risk score groups. P < 0.05 indicated a significant difference.

### Ethics approval and consent to participate

The GEO database is a public database. Users can download relevant data for free for research and publish relevant articles. Our study is based on open source data, so there are no ethical issues.

## Data Availability

The datasets for this study can be found in the Target (https://ocg.cancer.gov/programs/target) and GEO (https://www.ncbi.nlm.nih.gov/geo/query/acc.cgi?acc=GSE21257) databases.

## Abbreviations

ICD: Immunogenic cell death
OS: Osteosarcoma
RCD: Regulated cell death
TAA: Tumour-associated antigen
TSA: Tumour-specific antigen
DAMPs: Damage-associated molecular patterns
GEO: Gene Expression Omnibus
DEGs: Differentially expressed genes
GO: Gene Ontology
KEGG: Kyoto Encyclopedia of Genes and Genomes
RS: Risk score
KM: Kaplan‒Meier
HMGB1: High mobility group box 1
